# Childhood Self-Control Predicts Smoking Throughout Life: Evidence From 21,000 Cohort Study Participants

**DOI:** 10.1037/hea0000393

**Published:** 2016-09-08

**Authors:** Michael Daly, Mark Egan, Jody Quigley, Liam Delaney, Roy F. Baumeister

**Affiliations:** 1 Behavioural Science Centre, University of Stirling and UCD Geary Institute, University College Dublin; 2 Behavioural Science Centre, University of Stirling; 3 Division of Psychology, University of Stirling; 4 Behavioural Science Centre, University of Stirling and UCD Geary Institute, University College Dublin; 5 Department of Psychology, Florida State University

**Keywords:** personality, self-control, smoking, tobacco use, longitudinal research

## Abstract

***Objective:*** Low self-control has been linked with smoking, yet it remains unclear whether childhood self-control underlies the emergence of lifetime smoking patterns. We examined the contribution of childhood self-control to early smoking initiation and smoking across adulthood. ***Methods:*** 21,132 participants were drawn from 2 nationally representative cohort studies; the 1970 British Cohort Study (BCS) and the 1958 National Child Development Study (NCDS). Child self-control was teacher-rated at age 10 in the BCS and at ages 7 and 11 in the NCDS. Participants reported their smoking status and number of cigarettes smoked per day at 5 time-points in the BCS (ages 26–42) and 6 time-points in the NCDS (ages 23–55). Both studies controlled for socioeconomic background, cognitive ability, psychological distress, gender, and parental smoking; the NCDS also controlled for an extended set of background characteristics. ***Results:*** Early self-control made a substantial graded contribution to (not) smoking throughout life. In adjusted regression models, a 1-*SD* increase in self-control predicted a 6.9 percentage point lower probability of smoking in the BCS, and this was replicated in the NCDS (5.2 point reduced risk). Adolescent smoking explained over half of the association between self-control and adult smoking. Childhood self-control was positively related to smoking cessation and negatively related to smoking initiation, relapse to smoking, and the number of cigarettes smoked in adulthood. ***Conclusions:*** This study provides strong evidence that low childhood self-control predicts an increased risk of smoking throughout adulthood and points to adolescent smoking as a key pathway through which this may occur.

Self-control, or the ability to control impulses in the service of long-term goals, enables people to forgo tempting but unhealthy behaviors ranging from fatty food consumption to smoking and illicit substance use. The effortful self-governance that characterizes self-control draws together a broad set of constructs (e.g., effortful control, self-regulation, inhibitory control, willpower, delay of gratification, time discounting) that in turn have been linked with protective health-related behaviors (de Ridder, Lensvelt-Mulders, Finkenauer, Stok, & Baumeister, 2012; Duckworth, 2011; MacKillop et al., 2011; Smith, Mattick, Jamadar, & Iredale, 2014). Recent research suggests that self-control early in life leads to health benefits later. Children capable of inhibiting prepotent responses and abstaining from gratifying immediate desires tend to become healthier adults with lower body mass (Schlam, Wilson, Shoda, Mischel, & Ayduk, 2013), better general physical health, and lower rates of substance dependence (Moffitt et al., 2011).

The current research builds on this work. An emerging psychological literature demonstrates that individual differences in self-control predict smoking in adolescence (Audrain-McGovern et al., 2009; Audrain-McGovern, Rodriguez, Tercyak, Neuner, & Moss, 2006; deBlois & Kubzansky, 2015; Moffitt et al., 2011; Piehler, Véronneau, & Dishion, 2012) and adulthood (Bogg & Roberts, 2004; de Ridder et al., 2012). We tested the hypothesis that childhood self-control predicts early tobacco use and smoking across life.

## Self-Control and Smoking

Why are some children more vulnerable than others to becoming tobacco users? One factor is environmental exposure: being raised by parents who smoke or in an environment where smoking is prevalent has been linked to early smoking initiation (Bricker et al., 2006; Hiscock, Bauld, Amos, Fidler, & Munafo, 2012). Another factor is early personality. Enduring behavioral tendencies emerge early and have consequential effects on a range of adult outcomes. For example, the capacity to exert self-control over thoughts and actions from ages 3 to 11 predicts substance dependence at age 32 (Moffitt et al., 2011).

Longitudinal studies have also linked childhood and adolescent conscientiousness to subsequent smoking (e.g., Pluess & Bartley, 2015). In fact, a substantial part of the health benefits of conscientiousness comes from not smoking (Hampson, Edmonds, Goldberg, Dubanoski, & Hillier, 2015; Hampson, Goldberg, Vogt, & Dubanoski, 2007). Self-regulatory processes may underlie the development of conscientiousness (Eisenberg, Duckworth, Spinrad, & Valiente, 2014) and explain why more conscientious individuals tend not to smoke and live longer, healthier lives (Bogg & Roberts, 2004; Costa, Weiss, Duberstein, Friedman, & Siegler, 2014; Hampson et al., 2016; Turiano, Hill, Roberts, Spiro, & Mroczek, 2012).

Only a few longitudinal studies have shown that self-control problems place children at risk of subsequent smoking initiation in adolescence (deBlois & Kubzansky, 2015; de Winter, Visser, Verhulst, Vollebergh, & Reijneveld, 2015; King, Fleming, Monahan, & Catalano, 2011; Lee, McClernon, Kollins, Prybol, & Fuemmeler, 2013; Moffitt et al., 2011) and, in turn, increase risk of smoking in young adulthood (Lipkus, Barefoot, Williams, & Siegler, 1994; Welch & Poulton, 2009). The link between childhood self-control and smoking has not been examined using national data, nor has the potential protective role of self-regulatory skills in reducing the persistence of smoking across adulthood been identified. The paucity of data linking childhood self-control to adult smoking is surprising considering that recent reviews have implicated personality, behavioral, and neurobiological measures of impaired self-control in all stages of smoking, including initiation, maintenance, and relapse (Bloom, Matsko, & Cimino, 2014; MacKillop et al., 2011).

Self-control, an important resource for resisting cravings and avoiding temptation, is likely to be vital to understanding who begins, continues, and gives up smoking. Children with low self-control are more susceptible to tobacco advertising and the influence of peers who smoke (Audrain-McGovern et al., 2006; Piehler et al., 2012; Wills et al., 2010). Adolescents with better self-control are less likely to begin smoking as young adults (Audrain-McGovern et al., 2009), and less impulsive smokers (presumably those with more self-control) are more successful in achieving their smoking cessation goals (Ida, Goto, Takahashi, & Nishimura, 2011). Finally, interventions that increase self-control can reduce the risk of relapse among quitters (Muraven, 2010).

## Aims of the Present Study

Existing research strongly suggests a potential role for self-control in shaping smoking habits. However, this work has been limited by the use of non-nationally representative samples, short periods of follow-up, lack of multiwave data, and personality measures elicited after smoking initiation. We used two large cohort studies containing comprehensive data on tobacco use over a prolonged period spanning childhood to midlife.

We hypothesized that low self-control is a core reason why children take up smoking in adolescence and continue to smoke throughout life. Furthermore, we aimed to address four key limitations of the previous literature. First, we used childhood measures of self-control to predict later smoking and, where possible, eliminated from the sample any children who were already smokers at baseline. Other studies measuring smoking and self-control at the same time introduce potential ambiguities because self-control can change in response to prolonged exposure to nicotine, smoking deprivation, and cessation (Bloom, Matsko, & Cimino, 2014; Ida et al., 2011; Sutin et al., 2013; Yamane et al., 2013). Second, we examined the potential confounding role of important contextual factors including low social class, parental smoking, and early individual differences including psychological distress and low cognitive ability, which increase the risk of tobacco use and covary with self-control (Bricker et al., 2006; Hiscock et al., 2012; Lynskey & Fergusson, 1995). Third, we prospectively examined smoking status across multiple life stages, allowing us to test whether adolescent smoking was a pathway from early life self-control to adult smoking. Fourth, by examining changes in smoking status across adulthood, we could test whether self-control underlies the processes that shape population smoking levels: smoking initiation, relapse, and cessation.

## Method

### Participants

This study used data from two nationally representative ongoing British birth cohort studies. The British Cohort Study (BCS) is a multidisciplinary prospective study of individuals born in Britain in a single week in 1970 and traced longitudinally to 2012. The National Child Development Study (NCDS) is a longitudinal study of children born in a single week in Britain in 1958, with the most recent wave of follow-up completed in 2013. All datasets used are listed in the Supplementary Materials, Section 1.

#### British Cohort Study

The BCS tracks individuals’ health, education, social development, and economic experiences across life. Follow-up assessments have been conducted in several waves from childhood through to adulthood. We used the BCS data to examine links between childhood self-control at age 10 and smoking behavior at ages 26, 30, 34, 38 and 42, using a sample of 8,526.

#### National Child Development Study

We used the NCDS to extend our analysis to an older cohort. We also used the richer background data available in the NCDS to more stringently test the contribution of childhood self-control, measured at ages 7 and 11, to smoking behavior at ages 23, 33, 42, 46, 50, and 55, using a sample of 12,605.

### Measures

#### Childhood self-control

In the BCS, self-control was measured at age 10 using 9 teacher-rated items based on questionnaires developed by Conners (1969) and Rutter (1967). Teachers rated the child’s typical ability to control attention (e.g., “pays attention in class,” “cannot concentrate on a particular task”) and persevere on tasks (e.g., “shows perseverance,” “fails to finish tasks”)—two core elements of common self-control measures (e.g., Hagger, Wood, Stiff, & Chatzisarantis, 2010)—using a visual analogue scale ranging from “not at all” to “a great deal” (coded numerically as 1 to 47). We reverse-scored ratings as appropriate so that higher scores always meant better self-control, and took the average of the nine items to obtain a composite self-control score (*M* = 31.3, *SD* = 10.1; Cronbach’s alpha = .92).

In the NCDS, self-control was measured at ages 7 and 11 using 13 teacher-rated items from the Bristol Social Adjustment Guides. These items described “impulsive acting out without regard for consequences” and included measures of attentional control and impulsive behavior (e.g., “cannot attend or concentrate for long” and “constantly needs petty correction”; Stott, 1969). Teachers underlined the phrases they thought described the child’s typical behavior; each underlined item was scored as 1 point. Items were reverse-scored so that higher scores indicated better self-control and total self-control scores were derived from the number of statements endorsed, for a maximum score of 13 points. We took the average of the age 7 and 11 scores (*M* = 11.6, *SD* = 1.7). If a participant had complete data for only one of the two time-points, we used that score. Although individual items from the NCDS scales were not available in the original NCDS data, high levels of reliability (Cronbach’s alpha = .87) were found in the validation study reported in Daly, Delaney, Egan, and Baumeister (2015). The validation exercise in Daly et al. (2015) also found that the self-control measures in both studies corresponded closely (*r* > .7 unadjusted correlation; *r* > .8 after adjustment for measurement error), with parents’ ratings of their children’s self-control on two contemporary self-control measures: the Brief Self-Control Scale (Tangney, Baumeister, & Boone, 2004) and the Domain-Specific Impulsivity Scale (Tsukayama, Duckworth, & Kim, 2013). Individual items and details of the scales are in the Supplementary Materials, Section 2.

Self-control measures in both studies were standardized to have a mean of 0 and a standard deviation of 1. Due to clustering at the high end of the scale, the maximum observed score in the NCDS was 0.8 *SD* above the mean.

#### Adult smoking

**Figure 1 fig1:**
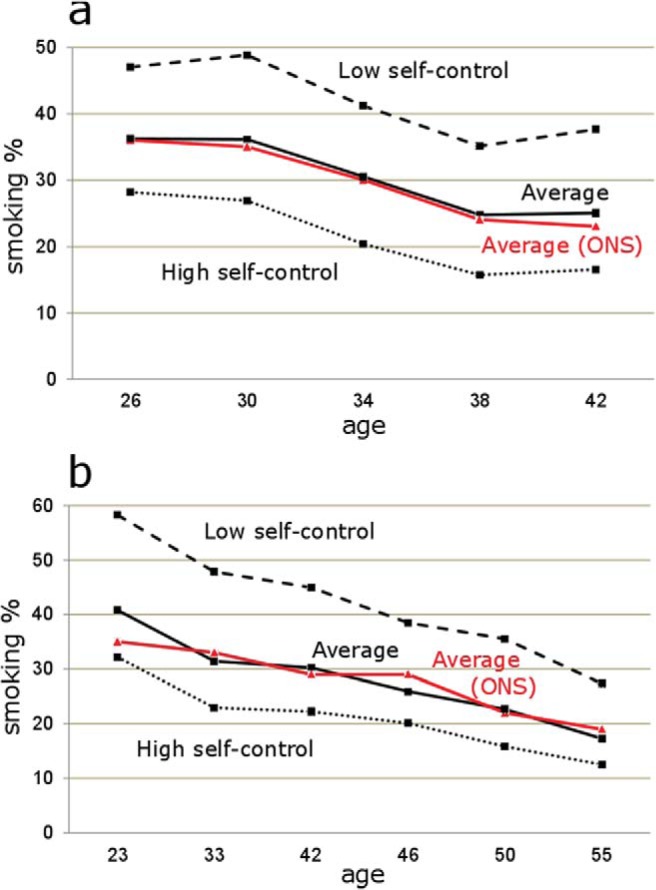
Percentage of smokers (daily and occasional combined) over time in the British Cohort Study (a) and the National Child Development Study (b). Average smoking levels in the cohort studies (black lines) are age and time-period matched to smoking statistics from the U.K. Office for National Statistics (ONS; red [dark gray] lines). Low self-control refers to cohort members scoring 1-*SD* and below the average self-control score (broken line); high self-control refers to those scoring 1-*SD* and above in the BCS, and 0.8-*SD* and above in the NCDS (dotted line). See Supplementary Materials, Section 3 for details of how ONS comparison figures were derived. See the online article for the color version of this figure.

In both cohorts, participants indicated whether they “smoke cigarettes every day,” “smoke cigarettes occasionally but not every day,” “used to smoke cigarettes but don’t at all now,” or “never smoked cigarettes” at each wave across adulthood. We created a categorical variable at each wave classifying participants as “never smokers,” “ex-smokers,” or “smokers” (daily and occasional smokers combined). Our “smoker” definition followed the U.K. Office for National Statistics (ONS) smoking classification system, which combines daily and occasional smokers, allowing smoking rates in the sample to be compared with national statistics (see Figure 1 and Supplementary Materials, Section 3). Our second outcome, also reported at each wave, examined the number of cigarettes smoked per day by daily smokers (i.e., those who reported they “smoke cigarettes every day”). The questions used to elicit both smoking outcomes are described in the Supplementary Materials, Section 4. Participants provided smoking data in 72.4% of possible survey waves, and a set of weighted analyses (available on request) showed that accounting for selection bias and the association between baseline characteristics and missing data across survey waves did not affect the relationship between self-control and smoking status.[Fig-anchor fig1]

In the BCS, it was possible to identify those who met the ONS criteria for child smoking at baseline—defined as a child who smokes at least one cigarette per week on average. To maintain clarity regarding the direction of influence between self-control and smoking behavior, we therefore removed 91 participants who reported smoking 1 or more cigarettes per week at age 10, when self-control was elicited.

#### Adolescent smoking

In order to test whether adolescent smoking mediated the relationship between childhood self-control and adult smoking behavior, we examined the number of cigarettes smoked per week at age 16 (where 1 = Nonsmoker; 2 = 1 cigarette; 3 = 2–10 cigarettes; 4 = 11–20 cigarettes; 5 = 21–40 cigarettes; 6 = 41+ cigarettes) in both cohorts. The questions used to derive our adolescent smoking measure are detailed in the Supplementary Materials, Section 4.

#### Parental smoking

All analyses adjusted for parental smoking, which was measured via parent-report when the cohort member was aged 10 in the BCS and 16 in the NCDS. Maternal and paternal smoking habits were coded as 0 = Nonsmoker; 1 = 1–10 cigarettes per day; 2 = 11–20 per day; 3 = 21+ per day; 4 = missing data. The NCDS also included a category for parental pipe/cigar smoking. Where information on maternal smoking was unavailable at age 16 in the NCDS, we used maternal smoking levels prior to pregnancy. See Supplementary Materials, Section 4 for the individual parental smoking items used.

#### Covariates

The other childhood covariates were gender, general cognitive ability, psychological distress, and social class. In the BCS, cognitive ability was measured at age 10 using the British Ability Scales (BAS), which consist of two verbal and two nonverbal tests (Elliott, Murray, & Pearson, 1978; Cronbach’s alpha = .93). In the NCDS, cognitive ability was measured at age 11 using 40 verbal and 40 nonverbal items (Pigeon, 1964; Cronbach’s alpha = .94). Psychological distress was measured at age 10 in the BCS using 5 teacher-rated items from the Neuroticism/Anxiety subscale of the Child Developmental Behaviors scale (Cronbach’s alpha = .85). In the NCDS, distress was measured at ages 7 and 11 using a teacher-rated measure of psychological distress (see Egan, Daly, & Delaney, 2015) for further details, and individual distress items are included in Supplementary Materials, Section 2). Social class, elicited at birth and derived from the father’s occupation, was classified into five categories based on the Registrar General’s Social Classes: I = professional occupations; II = managerial and technical occupations; III = skilled occupations; IV = partly skilled occupations; V = unskilled occupations. Two additional categories were included to code for “Other” occupational categories (e.g., father unemployed/absent), and missing data.

In addition to these covariates, the comparatively richer background data available in the NCDS allowed us to include eight additional childhood variables that could have affected the association between early self-control and smoking. These variables were the cohort member’s race, family difficulties, household size, father’s age, and the presence of headaches/epilepsy, intellectual disability, psychiatric problems, and low birth weight. Details of the individual variables are described in the Supplementary Materials, Section 5.

Finally, we included three traits that are conceptually related to self-control in supplementary robustness tests. We included measures of child conduct problems and hyperactivity (Lynskey & Fergusson, 1995) and assessed whether conscientiousness at age 16 diminished the contribution of childhood self-control to adult smoking independently of smoking behavior at age 16. Details of the measures used are included in Supplemental Materials, Section 6.

### Statistical Methods

#### Smoking status throughout adulthood

We specified multinomial logit regressions (0 = never smoker; 1 = ex-smoker; 2 = smoker) to estimate the probability of smoking at ages 26 to 42 in the BCS and ages 23 to 55 in the NCDS (*Model 1*) and used the *margins* command in Stata (Long & Freese, 2014) to present our results in terms of percentage point changes in the probability of the outcome. Ordinary least squares (OLS) regressions were used to examine the number of cigarettes smoked per day among daily smokers over the same periods (*Model 2*). In all analyses standard errors were clustered by individual in order to account for repeated observations across the waves, and both models controlled for age in order to take into account the declining trend in smoking over the lifespan (evident in Figure 1).

$$\mathrm{Model}\;1:\;\mathrm{Smoking status}\;{\left(\mathrm{smoker}/\mathrm{ex-smoker}/\mathrm{never smoker}\right)}_{it}=\beta_{0i}+\beta_{1}\;{\mathrm{self-control}}_{i}+\beta_{2}\;{\mathrm{sex}}_{i}+\beta_{3}\;{\mathrm{cognitive ability}}_{i}+\beta_{4}\;{\mathrm{psychological distress}}_{i}+\beta_{5}\;{\mathrm{parental smoking}}_{i}+\beta_{6}\;{\mathrm{social class}}_{i}+\beta_{7}\;{\mathrm{age}}_{t}+\beta_{8}\;{\mathrm{extended controls}}_{i}\;(\mathrm{NCDS only})+\varepsilon_{it}$$

$$\mathrm{Model}\;2:\;{\mathrm{Cigarettes smoked per day}}_{it}=\beta_{0i}+\beta_{1}\;{\mathrm{self-control}}_{i}+\beta_{2}\;{\mathrm{sex}}_{i}+\beta_{3}\;{\mathrm{cognitive ability}}_{i}+\beta_{4}\;{\mathrm{psychological distress}}_{i}+\beta_{5}\;{\mathrm{parental smoking}}_{i}+\beta_{6}\;{\mathrm{social class}}_{i}+\beta_{7}\;{\mathrm{age}}_{t}+\beta_{8}\;{\mathrm{extended controls}}_{i}\;(\mathrm{NCDS only})+\varepsilon_{it}$$

#### Early smoking initiation

We tested the indirect effect of childhood self-control on later life smoking by adding our measure of adolescent smoking to *Model 1* and then using the *khb* command in Stata (Karlson, Holm, & Breen, 2012) to calculate the mediation effect. The *khb* procedure is suitable for examining outcomes measured repeatedly over time and where direct cross-model comparisons cannot be made because the outcome variable is noncontinuous. In the current study *khb* performs the necessary decomposition to allow the indirect pathway from self-control through an ordinal mediator (adolescent smoking) to a categorical outcome (smoker/ex-/never adult smoker) to be estimated.

#### Smoking initiation, relapse, and cessation in adulthood

We modified *Model 1* to test the link between childhood self-control and three patterns of tobacco use in adulthood: (a) smoking initiation, defined as being a never-smoker at one study wave and beginning tobacco use and reporting being a smoker or ex-smoker at the next wave; (b) relapse, defined as being an ex-smoker at one wave and a smoker at the next; and (c) smoking cessation, defined as being a smoker at one wave and an ex-smoker at the next. We modeled the association between self-control and initiation/relapse/cessation across all survey waves simultaneously in order to gauge the average link between self-control and each of these three patterns of tobacco use.

## Results

### Descriptive Statistics

Descriptive statistics and bivariate correlations among key variables are shown in Table 1. Average smoking rates were 30.7% in the BCS and 28.9% in the NCDS; smoking rates declined over time (from 36.2% at age 26 to 25.0% at age 42 in the BCS and from 40.8% at age 23 to 17.3% at age 55 in the NCDS) and closely tracked the ONS nationally representative age-matched rates for the U.K. population over the same periods, as shown in Figure 1. Across all waves, the average number of cigarettes smoked per day was 13.5 (*SD* = 7.2) in the BCS and 15.9 (*SD* = 7.9) in the NCDS. [Table-anchor tbl1]

Self-control correlated negatively with smoking (BCS: *r* = −.19; NCDS: *r* = −.20) and with the number of cigarettes consumed by smokers per day (*r* = −.13 in both studies). Figure 1 shows that cohort members with low self-control (the 16.1% of cohort members in the BCS and 11.8% in the NCDS with self-control scores equal to or lower than 1-*SD* below the mean) consistently had smoking rates around 20 percentage points higher than those with high self-control (21.2% of the sample in the BCS scoring equal to or higher than 1-*SD* above the mean, 30.8% of the sample in the NCDS scoring .8-*SD* or greater above the mean). In both studies, average self-control scores decreased in a graded way across the three outcome categories, and the difference in average self-control scores between never smokers and smokers was around 0.4 standard deviations (BCS: Never smoker self-control score = 33.5, Ex-smoker = 31.8, Smoker = 29.5, sample *SD* = 9.9; NCDS: Never smoker self-control score = 12.0, Ex-smoker = 11.7, Smoker = 11.3, Sample *SD* = 1.6).

### Smoking Status Throughout Adulthood

Table 2 shows the main regression results. In both cohorts, higher levels of childhood self-control predicted lower rates of adult smoking after adjustment for cognitive ability, psychological distress, gender, social class at birth, age, and parental smoking in both cohorts, as well as the extended set of controls in the NCDS. The marginal effects reported can be interpreted as predicted percentage point differences in the outcome smoking category relative to the base outcome of being a “never smoker.”[Table-anchor tbl2]

Across ages 26 to 42 in the BCS, a 1-*SD* increase in childhood self-control predicted a 2.7 percentage point lower probability of being an ex-smoker, and a 6.9 point lower probability of being a smoker. Similarly, in the NCDS, across ages 23 to 55, a 1-*SD* increase in self-control predicted a 2.1 percentage point lower probability of being an ex-smoker, and a 5.2 point lower probability of being a smoker. On average across both cohorts and across all study waves, our fully adjusted regression models predicted 36.5% of those with low self-control (1-*SD* below the average) to be smokers compared to 24.6% of those with high self-control (+1-*SD* in BCS, +0.8-*SD* in NCDS). In additional analyses, not shown here, we found that high self-control was predictive of a low prevalence of smoking in each adult wave in each cohort.

The magnitude of association between childhood self-control and smoking was strikingly large compared to the effect of other covariates. On average across both cohorts the decrease in smoking from a 1-*SD* increase in self-control was similar to the effect of a 3-*SD* increase in cognitive ability, and the effect of moving from high to low self-control (+1-*SD* in BCS/+0.8-*SD* in NCDS to −1-*SD*) on smoking (13.8 percentage point greater probability in the BCS, 9.7 points in the NCDS) was comparable to the effect of moving from having two non-smoking parents to having two parents who each smoked 11–20 cigarettes per day (12.9 point greater probability of smoking in the BCS, 9.7 points in the NCDS).

The robustness tests conducted in both studies, available in the Supplementary Materials, Section 6, found that self-control remained an important predictor of smoking behavior even after adjusting for hyperactivity levels, conduct problems, and conscientiousness (controlling for these traits reduced the self-control coefficients by approximately 10% on average across both studies).

Across both studies, higher self-control also predicted a reduction in the average number of cigarettes smoked daily (BCS: *b* = −0.330, *SE* = 0.146, *p* < .05; NCDS: *b* = −0.279, *SE* = 0.123, *p* < .05), as shown in the Supplementary Materials, Section 7. The magnitude of this effect was modest: Across both studies and all waves, cohort members with low self-control were predicted to smoke around 0.6 more cigarettes per day compared to those with high self-control.

### Adolescent Smoking

At age 16, 24.4% / 34.6% of the eligible sample in the BCS/NCDS reported smoking 1 or more cigarettes per week, and 12.7% / 21.3% reported smoking more than 20 cigarettes per week. Higher childhood self-control predicted lower smoking levels at age 16 in OLS regressions (BCS: *b* = −0.233, *SE* = 0.028, *p* < .001; NCDS: *b* = −0.404, *SE* = 0.019, *p* < .001), and adolescent smoking was a strong predictor of being a smoker in adulthood (BCS: *b* = 0.185, *SE* = 0.026, *p* < .001; NCDS: *b* **=** 0.176, *SE* = 0.015, *p* < .001), providing initial support for our prediction that adolescent smoking might explain the association between early self-control and adult smoking. Formal mediation analysis confirmed this prediction, as shown in Table 3. In the BCS/NCDS, 48.5% / 64.9% of the association between self-control and smoking was explained by differences in adolescent smoking. Decomposing this average mediation effect revealed large indirect effects of heavy adolescent smoking; smoking more than 40 cigarettes per week at age 16 explained 30.9% (*p* < .001) of the association between self-control and adult smoking in the BCS and 34.4% (*p* < .001) in the NCDS.[Table-anchor tbl3]

### Smoking Initiation, Relapse, and Cessation in Adulthood

Higher self-control predicted a lower probability of smoking initiation in both studies (1.5 percentage point lower probability in BCS, 0.8 point lower probability in NCDS), as shown in Table 4. The results for relapse were mixed; higher self-control predicted a lower chance of relapse in the BCS (1.5 percentage points), but this association was not significant in the NCDS. Conversely higher self-control predicted a higher chance of cessation in the NCDS (1.2 points), but this association was not significant in the BCS. Thus, the data suggest that better self-control helps adults avoid starting to smoke, and may help adults who have taken up smoking to quit and to refrain from smoking again after they have quit.[Table-anchor tbl4]

## Discussion

Childhood self-control was strongly predictive of adult smoking over four decades in two large population-based birth cohorts with over 21,000 participants. Children with low self-control had substantially higher rates of smoking, even decades later at age 55. The predictive strength of self-control exceeded that of cognitive ability and psychological distress, and could not be accounted for by these factors or other established predictors of smoking such as social class and parental smoking. This association was found in the 1970 BCS and 1958 NCDS birth cohorts and remained stable as the samples aged from young adulthood to midlife. To our knowledge, this is the first study to demonstrate the predictive power of childhood self-control in forecasting the emergence and maintenance of adult smoking levels using national data.

These findings underscore the influential role of childhood self-control in shaping the onset and progression of substance use (Mischel, Shoda, & Rodriguez, 1989; Moffitt et al., 2011). In both cohorts examined, teachers rated children on whether they could manage their attention rather than become distracted, and persist to complete long-lasting tasks rather than give up easily. In the NCDS, teachers also indicated whether children tended to adhere to rules, misbehave, or act carelessly. Taken together these behaviors are indicative of the capacity for self-control. Attentional control underlies the ability to suppress a dominant response to allow a subdominant response to be executed, a process that typifies self-control, as does the ability to inhibit impulsive behavior when doing so is situationally appropriate (Diamond, 2013). In both studies, it appears these inhibitory and attentional control capabilities stretched far beyond classroom behavior to shape how susceptible children were to the temptation of cigarettes throughout their lives.

It is well-established that the majority of smokers initiate their habit in their teens and that earlier smoking initiation predicts longer periods of smoking. Accordingly, our mediation analyses revealed that poor childhood self-control tended to precede adolescent tobacco use, which then led to adult smoking. This finding was remarkably consistent across both cohorts and was driven by those who smoked heavily in adolescence. These findings support previous research suggesting that children low in self-control may be particularly vulnerable to the temptation of tobacco products in adolescence (Audrain-McGovern et al., 2006; Wills et al., 2010), with potentially profound long-term effects, such as persistent smoking into midlife and tobacco-related health problems (Lipkus et al., 1994; Welch & Poulton, 2009).

Although the current research highlighted the importance of adolescent smoking as a key path to later smoking, many children successfully avoided taking up smoking in their teenage years only to become smokers as adults. We therefore sought to capitalize on the multiwave nature of the cohort study data to shed further light on how self-control may shape patterns of smoking across adulthood. We found that less self-controlled children who had never smoked by early adulthood remained more likely to become smokers in both cohorts, suggesting that low childhood self-control is a basis for lifelong vulnerability to becoming a smoker. Ex-smokers were more likely to relapse to become smokers if they had low childhood self-control, albeit only in the BCS cohort. Conversely, smokers had a higher rate of smoking cessation in adulthood if they had high childhood self-control in the NCDS cohort. These results imply that self-control may represent a common psychological process underlying each stage of smoking behavior, from initiation, to cessation, to relapse, which together shape population smoking levels.

### Strengths and Limitations

Our study has important strengths. We found a robust and replicable association between self-control and adult smoking across follow-up periods spanning 44 years in two large population-based cohort studies. Smoking rates in both samples closely tracked national U.K. rates, supporting the generalizability of the study findings. The rich data available on adolescent smoking habits allowed us to examine how early smoking operated as a pathway between childhood self-control and adult smoking. In the BCS cohort, we could eliminate baseline smokers at age 10 to clarify that the direction of influence in this study was from self-control to subsequent smoking. In the NCDS, we could extend our regression analyses to include a broader set of background covariates (e.g., household size, family difficulties, child health).

The current research was limited in several respects. We used self-control scales that have not been fully validated, and that focused chiefly on forms of self-control unrelated to appetitive control. Even so, these measures correlate strongly (*r* > .7, *r* > .8 adjusted for measurement error) with contemporary, fully validated self-control scales (Daly et al., 2015; Tangney et al., 2004; Tsukayama et al., 2013). Future studies could incorporate such recent measures to assess parent and child ratings of self-control and supplement these with observer ratings and behavioral measures to reduce measurement error. We speculate that future replication studies using a more comprehensive account of self-control may reveal even stronger associations with smoking.

Our data are longitudinal but nonetheless correlational, and so it is difficult to assert that low childhood self-control causes subsequent smoking. Our supplementary regression analyses showed that the specific contribution of the self-control measures could not be attributed to child conduct problems, hyperactivity, or adolescent conscientiousness - constructs that overlap conceptually with self-control and that reliably predict smoking (e.g., Lynskey & Fergusson, 1995; Pluess & Bartley, 2015). Taken together, these findings suggest that early self-control is not likely to be acting as a proxy for individual differences in other related traits, although further work is needed to precisely single out the contribution of self-control to adult smoking.

The extent to which our findings are consistent across time periods and countries remains unclear. The strikingly similar results in both the BCS and NCDS cohorts, and the persistence of self-control in predicting smoking over time, suggest these linkages are time invariant. However, it is possible that recent global trends towards more stringent tobacco control legislation could have attenuated the impact of self-control. For example, previous research has shown that the introduction of a workplace smoking ban and large tax increases on tobacco have led to a reduction in heavy smoking among those with low self-control, the group typically most affected by immediate environmental contingencies (Daly, Delaney, & Baumeister, 2015). Further research is needed to decipher whether such measures can help break the link between low childhood self-control and smoking.

## Conclusions

Having good self-control by age 10–11 appears to form a powerful basis for avoiding tobacco use for many decades thereafter—indeed, as far as we can tell, throughout life. In contrast, children who lack self-control in the classroom tend to take up smoking in adolescence at higher rates than other children and continue to have an elevated risk of smoking for many decades. They smoke more, quit less often and less effectively, and relapse at higher rates than their more self-controlled peers when they do quit. Although our findings point to adolescence as a particularly important period when smoking habits may become established, those with low self-control who make it through adolescence without smoking are still more vulnerable to taking up smoking later on in adulthood.

It has long been thought that preventing children from taking up smoking can have lasting benefits. The public policy issue is therefore how to accomplish that. Many efforts focus on educating children about the dangers of smoking. The present findings suggested that these approaches may profitably be augmented by a quite different approach; namely, increasing general self-control. Although the present data do not speak to the viability of increasing children’s self-control, other work has explored ways of doing that (Diamond, 2012). Insofar as self-control is a domain-general capability (e.g., Muraven & Baumeister, 2000), improving it in any domain is likely to carry over into improved ability to avoid smoking.

Although early life may represent the most developmentally appropriate period for self-control training, there is also evidence that such training could also produce reductions in smoking and increases in smoking cessation in adulthood (Muraven, 2010; Oaten & Cheng, 2006). Smoking prevention interventions could be targeted at children or adolescents with low self-control, or they could be administered more broadly, with a strong focus on promoting self-control strategies so that those who need the most help will derive the most benefit (e.g., Brody, Kogan, Chen, & Murray, 2008; Chapman, Hampson, & Clarkin, 2014).

The findings of the current study suggest that integrating self-control into adolescent smoking prevention initiatives could produce lifelong health benefits. Prior work has indicated that raising a child with high self-control will improve his or her grades in school, educational attainment, employment prospects, popularity, quality of relationships, and mental and physical health (Daly et al., 2015; Mischel et al., 1989; Moffitt et al., 2011; Tangney et al., 2004). To that already formidable list we can add: not smoking — along with the diverse health and other benefits that nonsmokers enjoy.

## Supplementary Material

10.1037/hea0000393.supp

## Figures and Tables

**Table 1 tbl1:** Descriptive Statistics and Correlations for Key Variables in the British Cohort Study and the National Child Development Study

	BCS							NCDS						
Variables	%/*M* (*SD*)	SC	CA	PD	F	S	PS	%/*M* (*SD*)	SC	CA	PD	F	S	PS
Smoker^a^	30.7%	**−.19**	**−.12**	.02	**−.05**	**.09**	**.17**	28.9%	**−.20**	**−.18**	**.13**	**−**.02	**.11**	**.12**
Cigarettes per day^b^	13.5 (7.2)	**−.13**	**−.11**	.02	**−.17**	**.10**	**.16**	15.9 (7.9)	**−.13**	**−.12**	**.10**	**−.16**	**.12**	**.15**
Self-control	31.3 (10.1)	1	**.42**	**−.39**	**.20**	**−.14**	**−.15**	11.6 (1.7)	1	**.38**	**−.45**	**.26**	**−.14**	**−.11**
Cognitive ability	76.7 (14.3)	—	1	**−.22**	**−.05**	**−.29**	**−.19**	43.5 (16.0)	—	1	**−.38**	**−.07**	**−.28**	**−.13**
Psych. distress	18.8 (6.2)	—	—	1	**.05**	**.05**	**.04**	1.0 (1.2)	—	—	1	**−.12**	**.13**	**.09**
Female	51%	—	—	—	1	**−**.00	.01	49%	—	—	—	1	**−**.00	.02
Social class^c^	3.0 (.8)	—	—	—	—	1	**.19**	3.1 (.9)	—	—	—	—	1	**.14**
Parental smoking^d^	.82 (.88)	—	—	—	—	—	1	.86 (.87)	—	—	—	—	—	1
*Note.* Bolded correlations are significant at *p* < .01. SC = self-control; CA = cognitive ability; PD = psychological distress; F = female; S = social class; PS = parental smoking.														
^a^ “Smoker” is the average prevalence of smoking for all available waves. ^b^ “Cigarettes per day” takes the average number of cigarettes smoked for all available waves and is restricted to smokers only. ^c^ Social class at birth based on father’s occupational social class ranges from I (highest: professional/managerial occupations) to V (lowest: unskilled occupations) and excludes “other” and “missing” categories in order to include this variable in the correlation matrix. ^d^ “Parental smoking” takes the average of “father smoking” and “mother smoking” variables and was rated on a 0–3 scale where 0 = Not a smoker, 1 = 1–10 cigarettes per day, 2 = 11–20 per day, 3 = 21+ per day. It excludes “Pipes/cigars” and “missing” categories for the purpose of including this variable in the correlation matrix. If useable data was not available for both parents, we used data on one parent to maximize sample size.														

**Table 2 tbl2:** Childhood Self-Control Predicting Percentage Point Changes in Adult Smoking in the British Cohort Study (Age 26–42) and the National Child Development Study (Age 23–55)

	BCS		NCDS	
Variables	Ex-smoker	Smoker	Ex-smoker	Smoker
Self-control	−2.7*** (.5)	−6.9*** (.5)	−2.1*** (.4)	−5.2*** (.4)
Cognitive ability	2.0*** (.4)	−.7 (.5)	1.5*** (.4)	−3.0*** (.4)
Psych. distress	−2.1*** (.4)	−2.1*** (.5)	.3 (.4)	.5 (.4)
Female	3.4*** (.8)	−.5 (.9)	−2.2*** (.7)	2.6*** (.7)
Age	.8*** (.0)	−.9*** (.0)	.2*** (.0)	−.6*** (.0)
Paternal smoking				
Father non-smoker	—	—	—	—
Father 1–10 cigs	.8 (1.4)	4.2** (1.6)	.0 (1.1)	4.5*** (1.2)
Father 11–20 cigs	−1.8 (1.1)	7.5*** (1.3)	−1.1 (1.0)	6.4*** (1.1)
Father 21+ cigs	.8 (1.4)	10.8*** (1.7)	−3.0* (1.3)	7.4*** (1.5)
Father pipes/cigar	—	—	.014 (.014)	2.8 (1.5)
Maternal smoking				
Mother non-smoker	—	—	—	—
Mother 1–10 cigs	.9 (1.2)	4.1** (1.4)	−.3 (.9)	2.6** (.9)
Mother 11–20 cigs	−4.3*** (1.0)	5.4*** (1.3)	−5.2*** (.8)	3.3*** (1.0)
Mother 21+ cigs	−8.0*** (1.7)	10.2*** (2.5)	−2.1 (1.6)	4.7** (1.8)
Mother pipes/cigar	—	—	15.5 (11.0)	11.0 (12.7)
Extended controls^a^	N	N	Y	Y
*N*	8,526	8,526	12,605	12,605
Observations	30,888	30,888	54,775	54,775
*Note.* Columns contain marginal effects calculated after multinomial logit regressions clustered by the individual participant identifier and controlling for social class. The base outcome for all columns is “Never smoked.” Self-control, cognitive ability, and psychological distress are standardized (*M* = 0, *SD* = 1). “Non-smoker” is the base category for the parental smoking variables. Table omits “missing” categories for parental smoking variables, but these are included in the regression. Robust standard errors in parentheses.				
^a^ Eight childhood background variables described in Supplementary Materials, Section 5.				
* *p* < .05. ** *p* < .01. *** *p* < .001.				

**Table 3 tbl3:** Decomposition of the Total Effect of Childhood Self-Control on Adult Smoking Via the Indirect Effect of Adolescent Smoking Initiation in the British Cohort Study (Age 26–42) and the National Child Development Study (Age 23–55)

	BCS		NCDS	
	Coefficient (*SE*)	*p* value	Coefficient (*SE*)	*p* value
Total effect	−.561 (.060)	<.001	−.559 (.038)	<.001
Direct effect	−.289 (.057)	<.001	−.197 (.038)	<.001
Indirect effect	−.272 (.052)	<.001	−.362 (.031)	<.001
	BCS		NCDS	
	Mediation effect	*p* value	Mediation effect	*p* value
Cigs. per week at age 16				
None	—	—	—	—
1	.7%	.65	1.6%	.06
2–10	6.0%	.06	2.6%	.09
11–20	6.1%	.08	7.7%	<.001
21–40	4.8%	.41	18.6%	<.001
41+	30.9%	<.001	34.4%	<.001
Total mediation effect	48.5%	<.001	64.9%	<.001
*N*	3,683		9,553	
Observations	14,645		42,490	
*Note.* Mediation analyses are clustered by individual participant identifier and control for age, gender, cognitive ability, psychological distress, parental social class, and parental smoking habits. Top part of table presents multinomial logit coefficients produced using the *khb* method. Bottom part of table presents mediation effect of childhood self-control on adult smoking by levels of adolescent smoking intensity.				

**Table 4 tbl4:** Childhood Self-Control Predicting Percentage Point Changes in Adult Smoking Initiation, Relapse, and Cessation in the British Cohort Study (Ages 26–42) and the National Child Development Study (Ages 23–55)

	BCS			NCDS		
Model	Initiation	Relapse	Cessation	Initiation	Relapse	Cessation
Self-control	−1.5*** (.3)	−1.5* (.8)	.8 (.7)	−.8** (.3)	−.2 (.4)	1.2* (.5)
Extended controls^a^	N	N	N	Y	Y	Y
*N*	3,749	2,361	3,153	5,479	4,928	4,826
Observations	10,560	4,471	6,849	18,459	10,464	12,251
*Note.* Columns contain Probit marginal effects coefficients clustered by individual participant identifier and controlling for age, gender, cognitive ability, psychological distress, parental social class, and parental smoking habits. Self-control is standardized (*M* = 0, *SD* = 1). Robust standard errors in parentheses.						
^a^ Eight childhood background variables described in Supplementary Materials, Section 5.						
* *p* < .05. ** *p* < .01. *** *p* < .001.
