# Supplementary material for: Childhood Self-Control Predicts Smoking Throughout Life: Evidence From 21,000 Cohort Study Participants
Source: Health Psychol. 2016 Sep 8;35(11):1254–63. doi: 10.1037/hea0000393 (PMC5067157; doi:10.1037/hea0000393)
Supplement: Supplementary file 1 [file zg1008163316so1.doc]

**Supplemental Materials**

**Childhood Self-Control Predicts Smoking Throughout Life: Evidence from 21,000 Cohort Study Participants**

**by M. Daly et al., 2016, *Health Psychology***

**http://dx.doi.org/10.1037/hea0000393**

**Supplementary Information for:**

Childhood self-control and smoking throughout the lifespan: Evidence from 21,000 cohort study participants

**Section 1:** Details of data-sets used.

**Section 2:** Details of the BCS and NCDS self-control and psychological distress scales.

**Section 3:** Procedure for generating figures comparing smoking rates in the BCS and NCDS cohort studies with the national ONS UK rates in Figure 1.

**Section 4:** Questions used to elicit smoking behaviour.

**Section 5:** Extended controls included in the NCDS smoking models.

**Section 6:** Regressions controlling for childhood conduct problems, childhood hyperactivity, adolescent conscientiousness.

**Section 7:** Regressions (with covariates) showing childhood self-control predicting the number of cigarettes smoked per day in the BCS and NCDS.

**Section 1:** Details of data-sets used.

The data collection for the BCS and NCDS studies is coordinated by the University of London, Institute of Education, and Centre for Longitudinal Studies. All datasets are available through the UK Data Service website (<http://ukdataservice.ac.uk/>), where they were accessed for the purposes of the current study.

From the British Cohort Study, we used the following data-sets: Birth and 22-Month Subsample, 1970-1972 [SN2666], Ten-Year Follow-Up, 1980 [SN3723], Sixteen-Year Follow-Up, 1986 [SN3535], Twenty-Six Year Follow-Up, 1996 [SN3833], Thirty Year Follow-Up, 2000 [SN5558], Thirty-Four Year Follow-Up, 2004 [SN5585], Thirty-Eight Year Follow-Up, 2008 [SN6557], and Forty-Two Year Follow-Up, 2012 [SN7473].

From the National Child Development Study, we used the Childhood Data, Sweeps 0-3, 1958-1974 [SN5565], Sweep 4, 1981, and Public Examination Results, 1978 [SN5566], Sweep 5, 1991 [SN5567], Sweep 6, 1999-2000 [SN5578], Sweep 7, 2004-2005 [SN5579], Sweep 8, 2008-2009 [SN6137] and Sweep 9, 2013 [SN7669].

**Section 2:** Details of the BCS and NCDS self-control and psychological distress scales used in our analyses.

**Childhood self-control**

In the British Cohort Study, self-control scores were based on teacher ratings of the 11-item Disorganised Activity subscale of the 53-item Child Developmental Behaviors questionnaire, administered when the child was 10 years old. This measure was designed for the British Cohort Study and includes items from the Conners Teachers Hyperactivity Rating Scale (Conners, 1969) and the Rutter Teacher Behavioral Scale B (Rutter, 1967). We omitted two items due to poor face validity (“Confused or hesitant”, “Shows lethargic/listless behavior”), which did not affect the reliability of the scale.

The questions used were:

1. Child is daydreaming. **R**
2. Cannot concentrate on particular task. **R**
3. Becomes bored during class. **R**
4. Shows perseverance.
5. Easily distracted. **R**
6. Pays attention in class.
7. Forget on complex tasks. **R**
8. Completes tasks.
9. Fails to finish tasks. **R**

**R** means the score was reverse coded. Each item was scored on a visual analogue score ranging from “not at all” to “a great deal” (coded numerically as scores of 1 to 47). Individual item scores were summed and averaged to create a total self-control score (M = 31.3, SD = 10.1, Cronbach’s α = 0.92, N = 8,527). This score was then standardized to have a mean of 0 and standard deviation of 1.

In the National Child Development Study, childhood self-control was measured at ages 7 and 11. At both ages teachers rated the child’s behavior using on a 13-item scale drawn from the British Social Adjustment Guide (Stott, 1969). Teachers were asked to consider whether a series of behaviors indicating “impulsive acting out without regard for consequences" applied to the child. Teachers underlined each phrase which applied to the child.

The questions used were:

1. Sometimes eager, sometimes doesn't bother (answering questions).
2. Constantly needs petty correction (classroom behavior).
3. Too restless to remember for long (effect of correction).
4. Cannot attend or concentrate for long (cannot sit still when read to or during broadcasts, plays with things under desk) (attentiveness).
5. Rough and ready, slapdash (standard) (manual).
6. In informal play starts off others in scrapping and rough play.
7. Does not know what to do with himself, can never stick at anything long (free activity).
8. Misbehaves when teacher is out of room (liking the limelight).
9. Careless, untidy, often loses or forgets books, pen (belongings).
10. Gets very dirty during day (care for appearance).
11. Slumps, lolls about (posture).
12. Foolish pranks when with a gang (nuisance).
13. Follower in mischief (nuisance)

The NCDS does not individual item scores for these questions - instead there is only a derived composite scale score. We first took the average of the age 7 and 11 total scale scores to create an aggregate self-control score, where a higher score meant worse self-control. If self-control scores were available for only one age, we used that measure. We then inverted the scores so that a higher score meant better self-control. Self-control scores ranged from 2 to 13 in our main sample (M = 11.6, SD = 1.7, N = 12,605) and were standardized to a mean of 0 and standard deviation of 1. Due to clustering on the scale, the maximum value of the standardized variable was 0.82 SD above the mean. Individual scale items were not available, so we draw from estimates of the internal reliability of the scale produced in a separate validation study reported elsewhere (Cronbach’s α = 0.87; Daly, Delaney, Egan, & Baumeister, 2015).

**Childhood psychological distress**

In the British Cohort Study, our measure of psychological distress was created from 5 teacher-rated items elicited when the cohort member was age 10. Each item was scored on a visual analogue scale from “none at all” to “a great deal” (ranging numerically from 1 to 47) and the scale showed high internal consistency (Cronbach’s α = 0.85). All scores were summed and averaged to create a composite distress score. Scores ranged from 1.2 to 38 in our main sample (M = 18.8, SD = 6.2, N = 8,527) and we standardized this variable to have a mean of 0 and standard deviation of 1.

The items were:

1. Afraid of new things / situations.
2. Behaves ‘nervously’
3. Fussy or over-particular
4. Worried and anxious
5. Anxious, worried

In the National Child Development Study, cohort members were assigned a ‘depression’ score at ages 7 and 11 by their teacher based on questions from the depression subscale of the British Social Adjustment Guide (Stott, 1969). The teachers were given the list of phrases below and asked to underline the items they thought described the child’s behavior or attitude. The underlined phrases were then summed to create the aggregate psychological distress score. We took the average of the age 7 and 11 scores, coded so that a higher score meant more distress. Scores ranged from 0 to 10 in our main sample for this variable (M = 1.0, SD = 1.2, N = 12,605). We then standardized this variable to have a mean of 0 and standard deviation of 1.

The questions used were:

1. Depends on how he feels (asking teacher's help).
2. Varies noticeably from day to day (persistence in class work).
3. Sometimes alert, sometimes lethargic in team games.
4. In free activity sometimes lacks interest.
5. Persistence in manual tasks varies greatly.
6. Impatient, loses temper with job (persistence - manual tasks).
7. Flies into a temper if provoked (physical prowess).
8. Can work alone but has no energy (persistence in class work).
9. Lacks physical energy (persistence manual tasks).
10. Has no life in him (class room behavior).
11. Apathetic (just sits) (attentiveness).
12. Shuffles restlessly (posture).
13. In asking teacher's help too apathetic to bother.
14. Dull listless eyes.
15. Always sluggish, lethargic in team games.
16. Sometimes wanders off alone (companionship).
17. Speech is thick, mumbling, inaudible.
18. Expression is miserable, depressed (under the weather) seldom smiles.

**Section 3:** Procedure for generating figures comparing smoking rates in the BCS and NCDS cohort studies with the national ONS UK rates in Figure 1.

The national smoking figures were obtained from the Office for National Statistics (ONS) Adult Smoking Habits in Great Britain survey, 1974-2013 (available at: <http://www.ons.gov.uk/ons/taxonomy/search/index.html?nscl=Smoking+Habits&nscl-orig=Smoking+Habits&content-type=Dataset&content-type=Reference+table&sortDirection=DESCENDING&sortBy=pubdate>), which contains data on the percentage of UK people across all age groups who report smoking, being an ex-smoker, and never having smoked, and the average number of cigarettes reportedly smoked per day. Data tables from the survey were used to match individuals of the relevant ages in each wave of the BCS and NCDS cohorts, to the equivalent aged national sample in that particular year. For example, in the BCS, participants were 30 years old in 2000, so to compare their smoking behavior with the corresponding national figure for this group, we used the figures for those who were 25-34 years old in 2000 from the ONS data. Likewise, participants of the NCDS were aged 55 in 2013, so the national figures for this group were obtained from those who were aged 50-59 in 2013 in the ONS survey.

**Section 4:** Questions used to elicit smoking behaviour.

Table S1 describes the four types of smoking variables used in our study: (i) the “parental smoking” variables recording information on the smoking habits of the cohort members’ father and mother at age 10 in the BCS and age 16 in the NCDS (due to missingness for maternal smoking at age 16 in the NCDS, this variable was supplemented with a measure recorded maternal pre-pregnancy smoking behaviour) (ii) the “adolescent smoking” measures recording the cohort members’ own smoking behaviour at age 16 in both studies (iii) the “smoking status” and “cigarettes smoked per day” measures elicited at ages 26, 30, 34, 38 and 42 in the BCS and ages 23, 33, 42, 46, 50, 55 in the NCDS which serve as our two main outcome variables. In our analysis “smoking status” was coded into three categories: “Never smoker”, “Ex-smoker” and “Smoker”, the last of which combined the “occasional” and “daily” smoker categories shown elicited in Table S1. “Cigarettes smoked per day” was elicited in some waves for occasional and daily smokers, in others for daily smokers only - in order to maintain consistency we restricted our analysis of this outcome to daily smokers only

Table S1

*Description of parental, adolescent, and adult smoking variables elicited in both studies.*

| **British Cohort Study (BCS)** | | | | |
| --- | --- | --- | --- | --- |
|  | Age | Variable description | Source of var. description | Variable name |
| Parental smoking | 10 | “Does the [mother/father] smoke cigarettes or cigars at present? If so, how many cigarettes does [he/she] smoke per day?” | a3723udb.pdf, p44 | e9_1 (mother), e11_1 (father) |
| Adolescent smoking | 16 | “How many cigarettes do you smoke in a week? I am a non smoker / I probably smoke one whole cigarette a week on average / More than 1 and up to 5 / More than 5 and up to 10 / More than 10 and up to 20 / More than 20 and up to 40 / More than 40 and up to 70 / More than 70 and up to 100 / More than 100 a week on average.” | a3535uab.pdf, p171 | gh2 |
| Smoking status | 26 | “Which of the following describes your smoking habit? I’ve never smoked cigarettes / I used to smoke cigarettes but don’t at all now / I now smoke cigarettes occasionally but not every day / I smoke cigarettes every day.” | b3833uab.pdf, p51 | b960632 |
| Cigarettes per day | 26 | IF YOU DO SMOKE: “How many [cigarettes] do you usually smoke in a day?” | “ | b960633 |
| Smoking status | 30 | “Would you say that you’ve never smoked cigarettes / you used to smoke cigarettes but don’t at all now / you now smoke cigarettes occasionally but not every day / you smoke cigarettes every day?” | user_guide_1999-2000_vol_2_capi_documentation.pdf, p190 | smoking |
| Cigarettes per day | 30 | IF SMOKE EVERYDAY: “How many cigarettes a day do you usually smoke?” | “ | nofcigs |
| Smoking status | 34 | Would you say that “you’ve never smoked cigarettes / you used to smoke cigarettes but don’t at all now / you now smoke cigarettes occasionally but not every day / you smoke cigarettes every day”? | bcs_2004_follow-up_capi_questionnaire.pdf, p142 | bd7smoke |
| Cigarettes per day | 34 | IF SMOKE EVERYDAY: “How many cigarettes a day do you usually smoke?” | “ | b7nfcigs |
| Smoking status | 38 | “Would you say that you’ve never smoked cigarettes / you used to smoke cigarettes but don’t at all now / you now smoke cigarettes occasionally but not every day / you smoke cigarettes every day?” | bcs_2008_follow-up_cati_questionnaire.pdf, p173 | b8smokig |
| Cigarettes per day | 38 | IF SMOKE EVERYDAY: “How many cigarettes a day do you usually smoke?” | “ | b8nfcigs |
| Smoking status | 42 | “Which of the statements on the card applies to you? I’ve never smoked cigarettes / I used to smoke cigarettes but don’t at all now / I now smoke cigarettes occasionally but not every day / I smoke cigarettes every day.” | bcs70_2012_follow_up_questionnaire_documentation.pdf, p174-75 | b9smokig |
| Cigarettes per day | 42 | IF SMOKE EVERYDAY: “How many cigarettes a day do you usually smoke?” | “ | b9nfcigs |
|  |  |  |  |  |
|  |  |  |  |  |
| **National Child Development Study (NCDS)** | | | | |
|  | Age | Variable description | Source of var. description | Variable name |
| Parental smoking | 0 / 16 | [Age 0] “Did the patient smoke as many as one cigarette during the 12 months before the start of the pregnancy? If so, how many per day during that period?”  [Age 16] “Number of cigarettes a day. Does not smoke / Occasional – less than 1 a day. 1-5. 6-10. 11-20. 21-30. 31 or more. Smokes pipe or cigars only.” | pms_1958_questionnaire.pdf, p2 (pre-pregnancy smoking) p172ncds3_1974_questionnaires_and_codebook.pdf, p172 (parental smoking at age 16) | n502 (mother, pre-pregnancy), n2400 (mother, age 16), n2401 (father, age 16) |
| Adolescent smoking | 16 | “How many cigarettes do you usually smoke in a week? None, don’t smoke / Less than 1 a week / Between 1 and 9 a week / Between 10 and 19 a week / Between 20 and 29 a week / Between 30 and 39 a week / Between 40 and 49 a week / Between 50 and 59 a week / 60 or more a week.” | “, p227 | n2887 |
| Smoking status | 23 | Smoking status was originally derived from multiple questions - we used a derived variable produced by the NCDS staff which categorizes these responses into one variable coded as “Never smoked/Ex-smoker/Current l-9 [cigarettes per day]/ Current 10-19/Current 20-29/Current 30-39/Current 40+.” | ncds4_1981_part_1_data_dictionary_questionnaires_showcards.pdf, p115-16 | currentn |
| Cigarettes per day | 23 | “How many cigarettes a day do you usually smoke? If varies, take average over a week.” | “, p115 | n5935 |
| Smoking status | 33 | We combined two questions to create our three category variable. “Never smoker” and “ex-smoker” were derived from “IF DOESN’T SMOKE: Have you ever smoked cigarettes regularly – by regularly I mean at least one cigarette a day for 12 months or more?” Those answering “no” were classified as “Never smokers” and those answering “yes” were classified as “ex-smokers”. We classified “smokers” based on those who reported smoking in response to “How many cigarettes a day do you usually smoke?” | ncds5_1991_part_2_questionnaires.pdf, p135 | n504263, n504265 |
| Cigarettes per day | 33 | “How many cigarettes a day do you usually smoke?” | “ | n504263 |
| Smoking status | 42 | “Would you say that you’ve never smoked cigarettes / you used to smoke cigarettes but don’t at all now / you now smoke cigarettes occasionally but not every day / you smoke cigarettes every day?” | user_guide_1999-2000_vol_2_capi_documentation.pdf, p176 | smoking |
| Cigarettes per day | 42 | IF SMOKE EVERYDAY: “How many cigarettes a day do you usually smoke?” | “” | nofcigs |
| Smoking status | 46 | “Would you say that you’ve never smoked cigarettes / you used to smoke cigarettes but don’t at all now / you now smoke cigarettes occasionally but not every day / you smoke cigarettes every day?” | ncds_2004_cati_questionnaire_documentation.pdf, p91 | nd7smoke |
| Cigarettes per day | 46 | IF SMOKE EVERYDAY: “How many cigarettes a day do you usually smoke?” | “ | n7nfcigs |
| Smoking status | 50 | “Would you say that you’ve never smoked cigarettes / you used to smoke cigarettes but don’t at all now / you now smoke cigarettes occasionally but not every day / you smoke cigarettes every day?” | ncds8_final_mainstage_documentation.pdf, p225 | nd8smoke |
| Cigarettes per day | 50 | IF SMOKE EVERYDAY: “How many cigarettes a day do you usually smoke?” | “ | n8nfcigs |
| Smoking status | 55 | “Which of these statements applies to you? I’ve never smoked cigarettes / I used to smoke cigarettes but don’t at all now / I now smoke cigarettes occasionally but not every day / I smoke cigarettes every day.” | ncds_2013_follow_up_questionnaire_documentation.pdf, p178-79 | n9smokig |
| Cigarettes per day | 55 | IF SMOKE EVERYDAY: “How many cigarettes a day do you usually smoke?” | “ | n9nfcigs |

**Section 5:** Extended controls included in the NCDS smoking models.

Table S2 describes the eight childhood background variables which were included in our NCDS analysis. These variables were the cohort member’s race, family difficulties, household size, father’s age, and the presence of headaches/epilepsy, mental retardation, psychiatric problems, or low birth weight. Table S2 also describes the imputation procedure we used for each of these variables, which was relatively simple. For the categorical variables (the first six in the table), we added a “missing” category. For the continuous variables (the last two in the table), we imputed missing values with either the median or mean score among those who did report data for these variables. When including these now partially-imputed variables in our regression, we also included a dummy variable indicating whether or not the values for these variables were imputed (1 = yes).

Table S2

*Details of the extended control variables used in the NCDS.*

| Variable | Age | Description | Coding | N before imputation | Imputation procedure | N after imputation |
| --- | --- | --- | --- | --- | --- | --- |
| Family difficulties scale | 7 | An index created by summing 10 dummy variables indicating whether any of the following were present in the childhood home at age 7: death of the father, death of the mother, housing difficulties, financial difficulties, domestic tension, alcoholism, unemployment, physical illness, mental illness, other difficulties. | 0 = None,  1 = 1 difficulty,  2 = 2+ difficulties | 8819 | Add “missing” category (3786 obs) | 12605 |
|  |  |  |  |  |  |  |
| Headaches/epilepsy | 7/7 | A dummy variable scored as 1 if the cohort member was recorded as having frequent headaches or migraine at age 7 or epilepsy at age 7. | 1 = yes for either | 11321 | “ (1284 obs) | 12605 |
|  |  |  |  |  |  |  |
| Mental retardation | 7 | A dummy variable scored as 1 if the cohort member was recorded as having any level of mental retardation at age 7 (yes but no handicap / slight handicap / moderate handicap / severe handicap). | 1 = yes | 10864 | “ (1741 obs) | 12605 |
|  |  |  |  |  |  |  |
| Emotional maladjustment/ Psychiatric treatment | 7/11 | A dummy variable scored as 1 if the cohort member was recorded as having any level of emotional maladjustment retardation at age 7 (yes but no handicap / slight handicap / moderate handicap / severe handicap) or had received psychiatric/psychological treatment by age 11. | 1 = yes for either | 12103 | “ (502 obs) | 12605 |
|  |  |  |  |  |  |  |
| Race | 16 | A dummy variable coded as 1 if the mother reported the cohort member as being non-white at age 16 (African-Negroid/Indian-Pakistan/Other Asian/Mixed race/Other”. | 1 = Non-white | 9189 | “ (3416 obs) | 12605 |
|  |  |  |  |  |  |  |
| Low birth weight | Birth | A dummy variable scored as 1 if the mother reported the cohort members’ birth weight at less than 88 ounces. | 1 = < 88 ounces | 12022 | “ (583 obs) | 12605 |
|  |  |  |  |  |  |  |
| Household size | 7 | A continuous variable elicited from the parents at age 7 recording the number of people in the childhood home, which we converted to a categorical variable. | 0 = 1 -3 people, 1 = 4 people, 2 = 5 people, 3 = 6 people, 4 = 7+ people | 10940 | Replace missing values with median score of 2 (1665 obs) and create dummy variable where 1 = imputed scores | 12605 |
|  |  |  |  |  |  |  |
| Husband’s age | Birth | The age of the father when the cohort members was born | Continuous variable | 11668 | Replace missing values with mean score of 30.64 (937 obs) and create dummy variable where 1 = imputed scores | 12605 |

**Section 6:** Smoking status regressions controlling for childhood conduct problems, childhood hyperactivity, and adolescent conscientiousness.

This section describes two robustness tests of the association between childhood self-control and adult smoking. The first, adjusts for mother-rated measures of childhood conduct problems and childhood hyperactivity. As both of these constructs are conceptually similar to self-control (Barkley, 1997) the inclusion of these measures in our smoking regressions is an attempt to stringently isolate the specific contribution of self-control. We aimed to select items from the data to match those from the ‘conduct problems’ and ‘hyperactivity’ sections of the well-validated Strengths and Difficulties Questionnaire.

In the BCS conduct problems were gauged using seven items (e.g. “fights other children”, “destroys belongings”, “often tells lies”) completed at age 10. Five items were used to assess hyperactivity (e.g. “restless”, “squirmy”, “can’t settle”). The items were rated on a scale from 0 – 100 and both scales demonstrated satisfactory levels of internal consistency (Cronbach’s α > .7). The composite ‘conduct problems’ and ‘hyperactivity’ variables were created by taking the average of their component items.

In the NCDS three-items were used to assess conduct problems (i.e. “fights other children”, “generally destructive”, “disobedient”) and hyperactivity (i.e. “restless”, “squirmy”, “can’t settle”) at ages 7 and 11. Mothers rated whether each behavior “never”, “sometimes”, or “frequently” applied to their child. Items were scored accordingly and summed to produce a composite scale. The NCS items showed a lower level of reliability than in the BCS (Cronbach’s α ≈ .6). Conduct and hyperactivity scores were standardized to have a mean of 0 and a standard deviation of 1 in both cohorts.

As can be seen in Tables S3 and S4 below, controlling for conduct problems and hyperactivity had a minimal effect on the association between early life self-control and smoking / ex-smoking. On average including conduct problems and hyperactivity in the regression models diminished the association between self-control and smoking / ex-smoking by under 9%.

Table S3

*Childhood self-control predicting adult smoking in the British Cohort Study (age 26-42), with and without controls for childhood conduct problems and childhood hyperactivity.*

| Outcome | Ex-smoker | | Ex-Smoker | | | Smoker | | Smoker | |
| --- | --- | --- | --- | --- | --- | --- | --- | --- | --- |
| Self-control | | -0.029*** | | -0.028*** | -0.071*** | | -0.064*** | |  |
|  | | (0.005) | | (0.005) | (0.006) | | (0.006) | |  |
| Conduct problems | |  | | 0.006 |  | | 0.007 | |  |
|  | |  | | (0.005) |  | | (0.006) | |  |
| Hyperactivity | |  | | -0.001 |  | | 0.026*** | |  |
|  | |  | | (0.005) |  | | (0.006) | |  |
| N | | 7,267 | | 7,267 | 7,267 | | 7,267 | |  |
| Observations | | 26,697 | | 26,697 | 26,697 | | 26,697 | |  |

Columns contain marginal effects calculated after multinomial logit regressions clustered by the individual participant identifier and controlling for the variables described in *Model 1*. The base outcome for all columns is “Never smoked”. Self-control, conduct problems and hyperactivity are standardized (*M* = 0, *SD* = 1). Robust standard errors in parentheses.

*** p<0.001, ** p<0.01, * p<0.05

Table S4

*Childhood self-control predicting adult smoking in the National Child Development Study (age 23-55), with and without controls for childhood conduct problems and childhood hyperactivity.*

| Outcome | Ex-Smoker | | | Ex-smoker | Smoker | Smoker |
| --- | --- | --- | --- | --- | --- | --- |
| Self-control | | -0.020*** | -0.019*** | | -0.057*** | -0.048*** |
|  | | (0.005) | (0.005) | | (0.005) | (0.005) |
| Conduct problems | |  | -0.002 | |  | 0.006 |
|  | |  | (0.004) | |  | (0.005) |
| Hyperactivity | |  | 0.010* | |  | 0.032*** |
|  | |  | (0.004) | |  | (0.005) |
| N | | 9,752 | 9,752 | | 9,752 | 9,752 |
| Observations | | 42,992 | 42,992 | | 42,992 | 42,992 |

Columns contain marginal effects calculated after multinomial logit regressions clustered by the individual participant identifier and controlling for the variables described in *Model 1*. The base outcome for all columns is “Never smoked”. Self-control, conduct problems and hyperactivity are standardized (*M* = 0, *SD* = 1). Robust standard errors in parentheses.

*** p<0.001, ** p<0.01, * p<0.05

Our second robustness test added controls for adolescent conscientiousness and adolescent smoking. In the BCS conscientiousness was self-reported at age 16 using three items drawn from the ‘Knowing Myself’ questionnaire: “I am punctual”, “I am reliable”, and “I am responsible”. These statements were rated on a scale of (1) “Does not apply”, (2) “Applies somewhat”, (3) “Applies very much”. The average of these three items (M = 7.44, SE = 1.31, N = 2,993, Cronbach’s α = .62) have previously been shown to correlate reasonably strongly with conscientiousness assessed using standard items from the 50-item version of the International Personality Item Pool (IPIP) in a sample of 389 adults (r = .67, p < .001; Egan, Daly, Delaney, Boyce, & Wood, under review).

In the NCDS conscientiousness was self-reported at age 16 using three items previously used to gauge this personality trait (Pluess & Bartley, 2015). First, participants rated themselves on a continuum ranging from ‘1=Lazy’ to ‘5=Hardworking’. Next, they completed two items related to homework and schoolwork: “I get on with classwork” and “I never take work seriously” which were rated from ‘1=very true’ and ‘5=Not true at all’. To create our conscientiousness variable we took the average of these three scores where higher scores indicated greater conscientiousness (M = 3.57, SD = 0.86; N = 9,002, Cronbach’s α = .60). Conscientiousness measures in both studies were standardized to have a mean of 0 and a standard deviation of 1.

As shown in Tables S5 and S6, higher conscientiousness predicted lower rates of current and ex-smoking in both cohorts. Our test of whether conscientiousness at age 16 diminished the contribution of childhood self-control independently of contemporaneous smoking behavior, showed that the introduction of conscientiousness did not produce a substantial further reduction in the relationship between early life self-control and smoking /ex-smoking (13% reduction in statistically significant associations).

Table S5

*Childhood self-control predicting adult smoking in the British Cohort Study (age 26-42), with and without controls for adolescent conscientiousness and adolescent smoking.*

| Outcome | Ex-smoker | Ex-smoker | Ex-smoker | Smoker | Smoker | Smoker |
| --- | --- | --- | --- | --- | --- | --- |
| Self-control | -0.031*** | -0.022** | -0.019* | -0.053*** | -0.029*** | -0.027*** |
|  | (0.008) | (0.008) | (0.008) | (0.008) | (0.007) | (0.007) |
| Adolescent smoking |  | 0.121*** | 0.118*** |  | 0.135*** | 0.132*** |
|  |  | (0.009) | (0.009) |  | (0.008) | (0.007) |
| Conscientiousness |  |  | -0.017** |  |  | -0.014* |
|  |  |  | (0.006) |  |  | (0.006) |
| N | 2,993 | 2,993 | 2,993 | 2,993 | 2,993 | 2,993 |
| Observations | 11,948 | 11,948 | 11,948 | 11,948 | 11,948 | 11,948 |

Columns contain marginal effects calculated after multinomial logit regressions clustered by the individual participant identifier and controlling for the variables in *Model 1*. The base outcome for all columns is “Never smoked”. Self-control and conscientiousness are standardized (*M* = 0, *SD* = 1). Adolescent smoking is a categorical variable coded as 0 = Non-smoker, 1 = 1 cigarette per day, 2 = 2-10 cigarettes, 3= 11-20 cigarettes, 4 = 21 – 40 cigarettes, 5 = 41+ cigarettes. Robust standard errors in parentheses.

*** p<0.001, ** p<0.01, * p<0.05

Table S6

*Childhood self-control predicting adult smoking in the National Child Development Study (age 23-55), with and without controls for adolescent conscientiousness and adolescent smoking.*

| Outcome | Ex-smoker | Ex-smoker | Ex-smoker | Smoker | Smoker | Smoker |
| --- | --- | --- | --- | --- | --- | --- |
| Self-control | -0.019*** | -0.003 | 0.000 | -0.051*** | -0.021*** | -0.017*** |
|  | (0.005) | (0.005) | (0.005) | (0.005) | (0.004) | (0.004) |
| Adolescent smoking |  | 0.083*** | 0.081*** |  | 0.095*** | 0.090*** |
|  |  | (0.002) | (0.003) |  | (0.002) | (0.002) |
| Conscientiousness |  |  | -0.014*** |  |  | -0.028*** |
|  |  |  | (0.004) |  |  | (0.004) |
| N | 9,002 | 9,002 | 9,002 | 9,002 | 9,002 | 9,002 |
| Observations | 40,243 | 40,243 | 40,243 | 40,243 | 40,243 | 40,243 |

Columns contain marginal effects calculated after multinomial logit regressions clustered by the individual participant identifier and controlling for the variables in *Model 1*. The base outcome for all columns is “Never smoked”. Self-control and conscientiousness are standardized (*M* = 0, *SD* = 1). Adolescent smoking is a categorical variable coded as 0 = Non-smoker, 1 = 1 cigarette per day, 2 = 2-10 cigarettes, 3= 11-20 cigarettes, 4 = 21 – 40 cigarettes, 5 = 41+ cigarettes. Robust standard errors in parentheses.

*** p<0.001, ** p<0.01, * p<0.05

**Section 7:** Regressions (with covariates) showing childhood self-control predicting the number of cigarettes smoked per day in the BCS and NCDS.

Higher self-control predicted a reduction in the average number of cigarettes smoked daily, as shown in Table S7 below. The predicted number of cigarettes smoked per day exhibited a declining gradient with higher self-control in both studies.

Table S7

*Childhood self-control predicting cigarettes smoked per day in the British Cohort Study (age 26-42) and the National Child Development Study (age 23-55).*

| Study | BCS | NCDS |
| --- | --- | --- |
| Outcome | Cigarettes per day | Cigarettes per day |
| Self-control | -0.330* | -0.279* |
|  | (0.146) | (0.123) |
| Cognitive ability | -0.208 | -0.264* |
|  | (0.150) | (0.130) |
| Psych. distress | 0.053 | 0.116 |
|  | (0.127) | (0.119) |
| Female | -2.197*** | -2.217*** |
|  | (0.250) | (0.224) |
| Age | 0.075*** | 0.010 |
|  | (0.014) | (0.007) |
| *Paternal smoking* |  |  |
| Father non-smoker | - | - |
|  | - | - |
| Father 1-10 cigs | -0.534 | -0.370 |
|  | (0.445) | (0.386) |
| Father 11-20 cigs | 0.084 | 0.509 |
|  | (0.321) | (0.327) |
| Father 21+ cigs | 1.331** | 1.990*** |
|  | (0.430) | (0.417) |
| Father pipes/cigar | - | -0.348 |
|  | - | (0.505) |
| *Maternal smoking* |  |  |
| Mother non-smoker | - | - |
|  | - | - |
| Mother 1-10 cigs | -0.525 | -0.116 |
|  | (0.363) | (0.282) |
| Mother 11-20 cigs | 1.372*** | 1.252*** |
|  | (0.309) | (0.263) |
| Mother 21+ cigs | 2.145*** | 2.338*** |
|  | (0.612) | (0.488) |
| Mother pipes/cigar | - | -1.431 |
|  | - | (3.950) |
| Extended controls ^a^ | N | Y |
| N | 3,283 | 5,427 |
| Observations | 7,654 | 14,254 |

Columns contain OLS coefficients clustered by the individual participant identifier and controlling for social class. Table omits “missing” categories for parental smoking variables but these are included in the regression. Self-control, cognitive ability and distress are standardized (*M* = 0, *SD* = 1). Constant omitted. Robust standard errors in parentheses.

*** p<0.001, ** p<0.01, * p<0.05

**References**

Barkley, R. (1997). *ADHD and the Nature of Self-Control*. New York: Guildford Press.

Conners, C. (1969). A teacher rating scale for use in drug studies with children. *American Journal of Psychiatry*, *126*, 884-888.

Daly, M., Delaney, L., Egan, M., & Baumeister, R. (2015). Childhood self-control and unemployment over the life-span: Evidence from two British cohort studies. *Psychological Science*, *26,* 709-723.

Egan, M., Daly, M., Delaney, L., Boyce, C.J., & Wood, A. M. (under review). Pre-labor market conscientiousness predicts lower lifetime unemployment.

Pluess, M. & Bartley, M. (2015). Childhood conscientiousness predicts the social gradient of smoking in adulthood: A life course analysis. *Journal of Epidemiology and Community Health, 69*, 330-338.

Rutter, M. (1967). A children’s behavior questionnaire for completion by teachers: Preliminary findings. *Journal of Child* *Psychology and Psychiatry*, *8*, 1–11.

Stott, D. H. (1969). *The Social Adjustment of Children: Manual to the Bristol Social Adjustment Guides*. London, England: University of London Press.
